
JOURNAL INFORMATION
==============================
NLM Title Abbreviation: Biospektrum (Heidelb)
Journal ID: Biospektrum (Heidelb)
NLM Journal ID: 9615685

Biospektrum

ISSN: 0947-0867
EISSN: 1868-6249

ARTICLE INFORMATION
==============================
PMCID: PMC8852955
DOI: 10.1007/s12268-022-1699-4
Article ID: 1699
Article version: 1
Subjects: Karriere, Köpfe & Konzepte

Buchrezension zu: Handbuch Reisepharmazie 2021/22

Handbuch Reisepharmazie 2021/22 Medizinisch-pharmazeutische Beratung für privat und beruflich Reisende Christian Schönfeld295 S., Deutscher Apotheker Verlag, 2021. SC, 49,80 €. ISBN: 9783769276084

Seifert Roland seifert.roland@mh-hannover.de

grid.10423.34 0000 0000 9529 9877 Medizinische Hochschule, Hannover, Deutschland
Electronic publication date: 2022 Feb 13
Print publication date: 2022
Volume: 28
Issue: 1
First page: 112
Last page: 112
Copyright: © Der Autor 2022
License: Dieser Artikel wird unter der Creative Commons Namensnennung 4.0 International Lizenz veröffentlicht, welche die Nutzung, Vervielfältigung, Bearbeitung, Verbreitung und Wiedergabe in jeglichem Medium und Format erlaubt, sofern Sie den/die ursprünglichen Autor(en) und die Quelle ordnungsgemäß nennen, einen Link zur Creative Commons Lizenz beifügen und angeben, ob Änderungen vorgenommen wurden. Die in diesem Artikel enthaltenen Bilder und sonstiges Drittmaterial unterliegen ebenfalls der genannten Creative Commons Lizenz, sofern sich aus der Abbildungslegende nichts anderes ergibt. Sofern das betreffende Material nicht unter der genannten Creative Commons Lizenz steht und die betreffende Handlung nicht nach gesetzlichen Vorschriften erlaubt ist, ist für die oben aufgeführten Weiterverwendungen des Materials die Einwilligung des jeweiligen Rechteinhabers einzuholen. Weitere Details zur Lizenz entnehmen Sie bitte der Lizenzinformation auf http://creativecommons.org/licenses/by/4.0/deed.de.
License URL: https://creativecommons.org/licenses/by/4.0/

issue-copyright-statement: © The Author(s), under exclusive licence to Springer-Verlag GmbH Deutschland, ein Teil von Springer Nature 2022

==============================
Funding note

Open Access funding enabled and organized by Projekt DEAL.

Diese Rezension erscheint Open Access.

